# Abolishing HIV-1 infectivity using a polypurine tract-specific G-quadruplex-forming oligonucleotide

**DOI:** 10.1186/s12879-016-1713-x

**Published:** 2016-07-22

**Authors:** Maike Voges, Carola Schneider, Malte Sinn, Jörg S. Hartig, Rudolph Reimer, Joachim Hauber, Karin Moelling

**Affiliations:** Heinrich Pette Institute-Leibniz Institute for Experimental Virology, Martinistrasse 52, 20251 Hamburg, Germany; Department of Chemistry and Konstanz Research School Chemical Biology, University of Konstanz, Universitätsstrasse 10, 78457 Konstanz, Germany; German Center for Infection Research (DZIF), partner site, Hamburg, Germany; Institute of Medical Virology, University of Zurich, Gloriastrasse 32, 8006 Zurich, Switzerland; Max Planck Institute for Molecular Genetics, Ihnestrasse 63-73, 14195 Berlin, Germany

**Keywords:** Antivirals, Microbicide, HIV, RNase H, Infectivity, G-quadruplex

## Abstract

**Background:**

HIV is primarily transmitted by sexual intercourse and predominantly infects people in Third World countries. Here an important medical need is self-protection for women, particularly in societies where condoms are not widely accepted. Therefore, availability of antiviral microbicides may significantly reduce sexual HIV transmission in such environments.

**Methods:**

Here, we investigated structural characteristics and the antiviral activity of the polypurine tract (PPT)-specific ODN A, a 54-mer oligodeoxynucleotide (ODN) that has been previously shown to trigger the destruction of viral RNA genomes by prematurely activating the retroviral RNase H. The stability of ODN A and mutants thereof was tested at various storage conditions. Furthermore, antiviral effects of ODN A were analyzed in various tissue culture HIV-1 infection models. Finally, circular dichroism spectroscopy was employed to gain insight into the structure of ODN A.

**Results:**

We show here that ODN A is a powerful tool to abolish HIV-1 particle infectivity, as required for a candidate compound in vaginal microbicide applications. We demonstrate that ODN A is not only capable to prematurely activate the retroviral RNase H, but also prevents HIV-1 from entering host cells. ODN A also exhibited extraordinary stability lasting several weeks. Notably, ODN A is biologically active under various storage conditions, as well as in the presence of carboxymethylcellulose CMC (K-Y Jelly), a potential carrier for application as a vaginal microbicide. ODN A’s remarkable thermostability is apparently due to its specific, guanosine-rich sequence. Interestingly, these residues can form G-quadruplexes and may lead to G-based DNA hyperstructures. Importantly, the pronounced antiviral activity of ODN A is maintained in the presence of human semen or semen-derived enhancer of virus infection (SEVI; i.e. amyloid fibrils), both known to enhance HIV infectivity and reduce the efficacy of some antiviral microbicides.

**Conclusions:**

Since ODN A efficiently inactivates HIV-1 and also displays high stability and resistance against semen, it combines unique and promising features for its further development as a vaginal microbicide against HIV.

## Background

Infection with HIV-1 is a global pandemic that particularly affects Third World countries. HIV is transmitted primarily by sexual intercourse and in 2013 more than 35 million people globally were living with HIV. Over 70 % of infected subjects reside in Sub-Saharan Africa, with enormous medical and socioeconomic consequences for these societies. Although access to antiretroviral therapy (ART) in low- and middle-income countries is increasing, still too many people are beyond the reach of antiretroviral treatment. Moreover, use of condoms is frequently not accepted by men in some of these societies. Thus, development of novel microbicides is not only of urgent medical need, but would also empower women with a means for self-protection.

Eleven clinical microbicide studies, representing six candidate products, have failed over the last 20 years and not a single microbicide is currently publicly available. First generation microbicides acted as surfactants to disrupt viral membranes, block non-specific HIV entry, or inactivate HIV by decreasing the pH in the vagina [1–4]. Recently, more specific antiretroviral agents have been included in microbicide development, either blocking viral entry by interacting with the HIV-1 gp120 surface protein, or interfering with viral reverse transcriptase or integrase activity. For example, Tenofovir, a nucleoside-analogue reverse transcriptase inhibitor (NRTI), showed contradictory results in clinical testing [4–8]. Another microbicide candidate drug, the non-nucleoside reverse transcriptase inhibitor (NNRTI) Dapivirine, is currently undergoing phase III clinical testing, where the drug is delivered using an intravaginal ring [9, 10]. However, targeting the reverse transcriptase alone may not be sufficient for efficient HIV inhibition. Therefore, it may be beneficial for advanced antiretroviral microbicide development to simultaneously address various steps in the viral life cycle, possibly by using agents that possess multiple antiviral activities.

We previously described ODN A, a novel oligonucleotide-based HIV-1 inhibitor that targets the highly-conserved extended polypurine tract (PPT) of HIV-1 for subsequent RNase H-dependent degradation of the viral RNA genome in cell-free HIV-1 particles [11–13]. By specifically recognizing the PPT sequence, ODN A mimics the RNA-DNA hybrid that normally occurs during reverse transcription inside cells, which in turn triggers premature activation of the viral RT/RNase H heterodimer [14]. Consequently, ODN A drives the HIV genome into self-destruction. A series of previous studies demonstrated that ODN A is non-toxic, and moreover that it shows high antiviral potency in cell culture infection assays, and efficacy in several animal models [11–13, 15–17].

Besides its antiviral potency, the stability of an antiviral compound is of highest importance for developing a successful microbicide. Usage and storage of microbicides must be as easy as possible to achieve high acceptance and adherence, especially in Third World countries. More worryingly, it was recently discovered that human semen, particularly semen-derived amyloid fibrils, can enhance HIV infectivity while impairing antiviral efficacy of microbicides [18–21].

Here we focused on investigating the stability and structure of ODN A, and analyzing its antiviral activity, particularly in the presence of amyloid SEVI fibrils and human semen samples. Our physical analyses demonstrated that ODN A forms G-based DNA hyperstructures, also referred to as “DNA frayed wires” [22–26] of very high stability and solubility. Furthermore, ODN A revealed high antiviral potency in cell culture, even in the presence of synthetic SEVI, natural human semen, or an approved lubricant for human use. Together these data suggest that based on its antiviral potency and physical characteristics, ODN A may be a valuable component of future vaginal microbicides.

## Methods

### Oligodeoxynucleotides and non-denaturing polyacrylamide gel electorphoresis (PAGE)

**Fig. 1:**
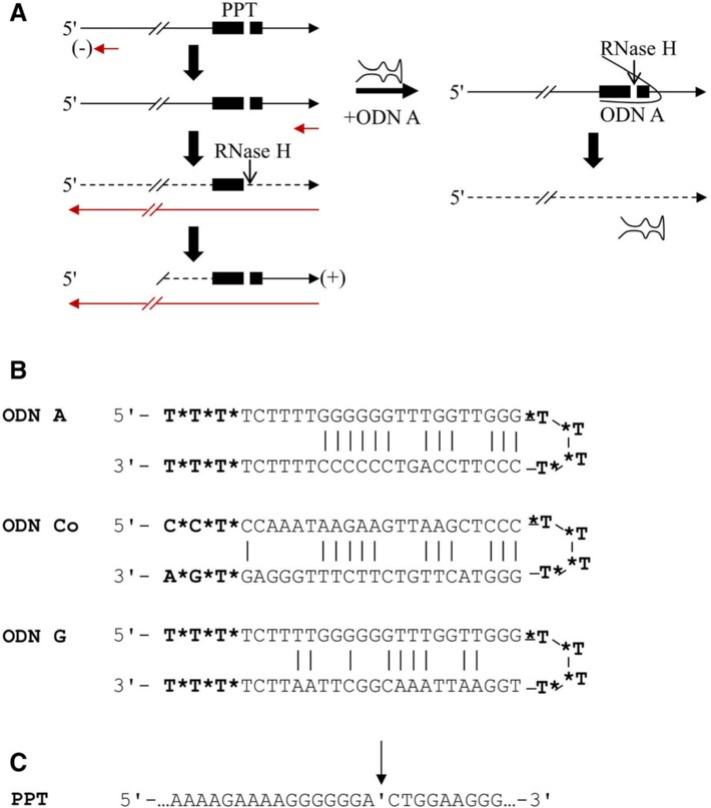
Antiviral mechanism of ODN sequences. **a** Schematic representation of reverse transcription of HIV and mechanism of ODN A action. The HIV-1 extended polypurine tract (PPT) is indicated as *black box* with the RT/RNase H cleavage side depicted in white. ODN A interacts with the highly conserved PPT, mimicking the natural replication intermediate RNA-DNA hybrid, which results in premature activation of reverse transcriptase (RT)/RNase H hydroloysis of the viral RNA genome. **b** Sequences of the ODNs used. All ODNs form hairpin-like structures with an antisense (*lower*) and passenger (*upper*) strand linked by four thymidines. Watson-Crick bonds are shown as *vertical bars*. Phosphorothioate-modified nucleotides are shown in *bold* and marked by a *star*. The ODN A sequence is complementary to the extended PPT, and ODN Co targets an HIV-1 RNA region outside of the PPT. ODN G serves as a further control, with a similar passenger strand sequence compared to ODN A but a non-complementary HIV-1 PPT sequence. **c** Sequence of the extended polypurine tract of HIV-1 recognized by the viral RNase H, whose specific cleavage site is indicated by an *arrow*

The ODNs (Integrated DNA Technologies, USA and Sigma-Aldrich Corporation, USA) comprise a 25-mer antisense and a 25-mer passenger strand, linked by four thymidines (Fig. 1). The ODN A sequence is partially complementary to the extended HIV-1 polypurine tract (PPT), ODN Co targets a region downstream of the PPT and, compared to ODN A, ODN G contains some nucleotide exchanges in the antisense strand to prevent binding to the HIV-1 PPT, whereas the passenger strand is identical (Fig. 1). ODN A: 5′- TTTTCTTTTGGGGGGTTTGGTTGGGTTTTCCCTTCCAGTCCCCCCTTTTCTTTT-3′; ODN Co: 5′-CCTCCAAATAAGAAGTTAAGCTCCCTTTTGGGTACTTGTCTTCTTTG GGAGTGA-3′; and ODN G: 5′- TTTTCTTTTGGGGGGTTTGGTTGGGTTTTCCCTTCC AGTCCCCCCTTTTCTTTT-3′. To increase their stability, all ODNs carry phosphorothioate modifications at three terminal and four central nucleotides [16]. The stability and formation of high molecular structures of the various ODNs were analyzed by non-denaturing 10 % PAGE followed by SYBR Green II staining.

### Cell culture and production of viral particles

HEK293T cells (ATCC; cat # CRL-3216) were cultured at 37 °C and 5 % CO_2_ in Dulbecco’s modified Eagle medium (DMEM, Biochrom, Germany) containing 10 % fetal calf serum (FCS, Biochrom, Germany). Jurkat 1G5 T cells (NIH AIDS Research & Reference Reagent Program; cat # 1819) were cultured in RPMI medium 1640 containing 10 % FCS (PAN-Biotech GmbH, Germany). Cellular assays were performed with Jurkat 1G5 T cells, which contain a stably integrated HIV-1 long terminal repeat (LTR)-firefly luciferase construct that is responsive to the HIV-1 Tat *trans*-activator protein. Replication-competent HIV-1 was produced by transfecting 3 × 10^6^ HEK293T cells with 10 μg of the pNL4-3mCherry plasmid using polyethylenimine (PEI) as a transfection reagent according to the manufacturer’s protocol (Polysciences, Inc., USA). The pNL4-3mCherry construct is a variant of the X4-tropic strain HIV-1_NL4-3_ [27], in which the *nef* gene was replaced by a sequence (711 bp) encoding the autofluorescent protein mCherry. At day 3 post transfection, virus-containing supernatants were passed through 0.2 μm pore size filters to ensure removal of any viral aggregates and kept at −80 °C. Titers of viral particles were determined by HIV-1 p24 antigen enzyme-linked immunosorbent assay (ELISA) as previously described [28].

### Synthetic RNA production and RT/RNase H cleavage assay

A plasmid containing a T7 promoter and the HIV-1 PPT sequence (synthesized by GeneArt AG, Germany) served as a template for producing synthetic PPT-containing RNA2 using the T7-Megashortscript Kit (Life Technologies GmbH, USA). The RNA2 sequence 5′-CTCGAGTAATACGACTCACTATAGGGAGAGGGAGGCAGCTGTAGATCTTAGCCACTTTT**AAAAGAAAAGGGGGGACTGGAAGGG**CTAATTCACTCCCAAAGAAGACAAGTACCCGGGATCGGTTAACGTCACACGTGCATGCGATATCGAATTC-3′ contains the binding site of ODN A (bold letters) and of ODN Co (underlined letters). Subsequently, the RNA2 in vitro transcript was dephosphorylated and radioactively 5′-labeled with γ-32-ATP (Hartmann Analytics, Germany) using the Kinase Max Kit (Life Technologies, USA). Afterwards, RNA2 transcripts were purified by 8 M UREA/10 % PAGE and gel elution for 16 h at 37 °C in elution buffer (0.5 M Ammonium Acid, 1 mM EDTA). For annealing, purified RNA2 transcripts were mixed with 50 nM of ODNs in hybridization buffer (50 mM NaCl, 10 mM MgCl_2_, 1 mM DTT, 0.4 mM spermine hydrochloride, 25 mM Tris-acetate, pH 6.8), heat-treated for 3 min at 90 °C, cooled, and incubated at 37 °C for 30 min. After annealing, samples were incubated with 0.05 units/μl of HIV RT/RNase H (Worthington, USA) for a further 30 min at 37 °C. The cleavage reaction was stopped by adding formamide RNA loading dye (New England Biolabs GmbH, USA), followed by incubation for 5 min at 90 °C. Cleavage was analyzed by denaturing 8 M UREA/10 % PAGE.

### Transmission electron microscopy of semen-derived enhancer of infection (SEVI)

Synthetic peptides corresponding to prostatic acid phosphatase (PAP) (European Molecular Biology Laboratory, AAB60640) amino acid residues 248–286 (PAP_248–286_) were obtained from Davids Biotechnologie, Germany and Bachem, Switzerland. Lyophilized peptides were resuspended in PBS at a stock concentration of 10 mg/mL, and aliquots were stored at −20 °C. Fibril formation by dissolved peptide (1 or 5 mg/mL) was initiated by agitation at 37 °C for 72 h by using an Eppendorf thermomixer. Negative staining of the fibrils was performed with 1 % Uranylacetat (Merck, Germany) on 400 mesh copper grids (Electron Microscopy Sciences, USA). Images were acquired with an OSIS Veleta CCD Camera attached to a FEI Technai G 20 Twin transmission electron microscope (FEI, Netherlands) at 80 kV.

### Semen samples

Human semen samples were donated by healthy volunteers, diluted 1:2 with PBS containing 100 units/mL penicillin, 100 μg/mL streptomycin, and 50 μg/mL gentamycin (Gibco), and stored at −20 °C.

### Infection of cells and viral load measurements

Prior to infection, 1 × 10^9^ HIV-1virions were pre-incubated with 250 nM of ODNs or buffer (1 mM sodium phosphate pH 8.0, 10 μM EDTA) in 100 μl RPMI + 10 % FCS for 6 h at 37 °C. 5 × 10^5^ cells were spinoculated at 230 × g in a sterile 15 mL falcon tube, supernatant was removed and the pellet was dissolved in virus/ODN mixture for 16 h in the presence of 2 μg/mL polybrene (Sigma-Aldrich Corporation, USA) (MOI 200). Next, cells were resuspended in 24-well plates using fresh RPMI + 10 % FCS medium containing 250 nM of ODNs or buffer. Every 3 to 4 days, supernatant was collected, fresh medium was supplemented with 250 nM ODNs or with buffer and cell numbers were determined. HIV-1 p24 antigen levels in the respective supernatants were determined by ELISA using a Versa Max Microplate Reader, Molecular Devices (USA). For cellular infection assays (containing SEVI or human semen samples), 5 × 10^8^ HIV-1 viral particles were pre-incubated with 250 nM of ODNs or buffer (1 mM sodium phosphate pH 8.0, 10 μM EDTA) and 100 μg/mL SEVI or human semen for 6 h at 37 °C. Human semen was thawed and 12.5 μl aliquots were diluted 1:16 in RPMI + 10 % FCS prior to infection. 5 × 10^5^ Jurkat 1G5 T cells were infected with the mixture as described before.

### In vitro HIV-1 fusion assay

HEK293T cells were transfected (TransIT, Mirus Bio LLC, USA) with HIV-1 Env and Tat expression vectors. Eight hours later, 5 × 10^5^ cells were transferred into 24-well plates and cultured overnight. Subsequently, 1 × 10^6^ Jurkat 1G5 T cells were incubated in RPMI + 10 % FCS supplemented with ODNs or buffer for 1 h at 37 °C. Afterwards, the medium of the transfected HEK293T cells was removed and Jurkat 1G5 T cells in medium supplemented with ODNs or buffer were added to the HEK293T cells. Twenty-four hours later, cells were lysed and luciferase activity was measured as relative light units per second (RLU/s) according to manufacturer’s protocol (Promega Corporation, USA) using a Centro LB960 (Berthold Technologies, Germany) reader.

### Statistical analysis

Statistical analysis was performed using Prism version 5.03 software (Graph Pad). The statistical significance was assessed by one-way or two-way analysis of variance (ANOVA) followed by a Dunnett’s Multiple Comparison Test or Bonferroni’s posttest. A result of *p* < 0.05 was considered to be statistically significant.

### Circular dichroism (CD) spectra

Five micrometre ODN A in buffer (Tris–HCl, 10 mM, pH 7.8 or pH 4.5 supplemented with annotated salts) was heated to 95 °C for 5 min and cooled slowly to RT. A CD spectrum was recorded with a Jasco J-815 CD spectrometer at 20 °C between 220 and 320 nm with data points every 0.5 nm. Scanning speed was set to 500 nm/min; bandwidth was 1 nm. The spectrum was measured five times and the mean was calculated for each wavelength. For melting CD spectra, 5 μM ODN A in buffer (Tris–HCl pH 7.5 supplemented with annotated salts) was heated to 100 °C for 5 min in the CD spectrometer. A spectrum from 220 to 320 nm was recorded as described before for every 5 °C decrease, from 100 to 20 °C. Spectra were depicted with MATLAB (The MathWorks, Inc, USA) software.

## Results

### ODN A is highly stabile at 37 °C and forms higher order molecular structures

Previously, we described a novel mechanism of viral RNA genome cleavage mediated by the viral RT/RNase H in cell-free viral particles. The active compound, the oligodeoxynucleotide (ODN) A, targets the sequence of the extended polypurine tract (PPT) of HIV-1, leading to premature activation of the retroviral RT/RNase H and hydrolysis of the HIV RNA genome (Fig. 1a) [11, 13, 29]. The sequence of the various ODNs analyzed here are depicted in Fig. 1b. ODN A comprises a 25-mer antisense strand targeted to the HIV-1 PPT and a 25-mer passenger strand connected by four thymidines. These thymidine residues (T4 linker) and the first three nucleotides at each terminus of the respective ODN are also modified with phosphorothioates to enhance stability. Due to partial complementarity of the antisense and passenger strand, the ODNs create a hairpin-like structure. However, ODN A directly targets the extended PPT, whereas the negative control, ODN Co, binds to sequences downstream of the extended PPT. The sequence of another control, ODN G, is identical to ODN A in the passenger strand with some nucleotide exchanges in the antisense strand to avoid binding to the viral RNA genome (see Fig. 1b). The extended HIV-1 PPT is characterized by two non-purines next to which the RNase H cuts in a highly specific manner, indicated with an arrow (Fig. 1c).

**Fig. 2:**
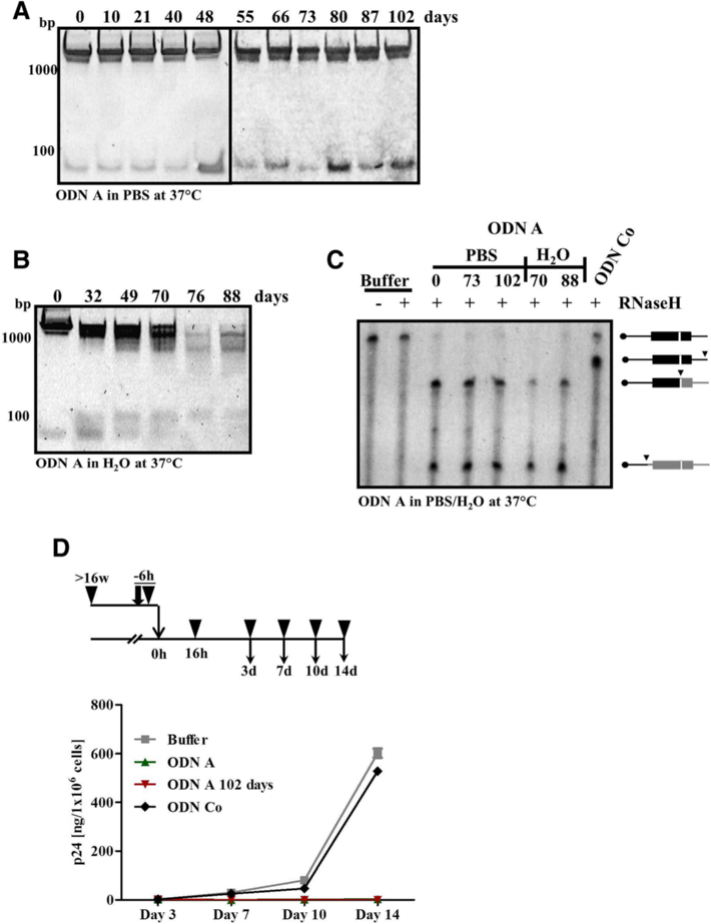
ODN A is active in vitro and in cell culture infection assays after long-term storage at 37 °C. **a** ODN A (8 μM) was stored for the indicated duration in PBS or (**b**) in H_2_O at 37 °C. Samples were analyzed by non-denaturing 10 % PAGE. **c** ODN A (50 nM), either freshly thawed or stored for the indicated time periods at 37 °C in PBS or H_2_O was hybridized to 50 nM in vitro transcribed γ-32-ATP 5′-labeled PPT-containing RNA in the presence of HIV-1 RT/RNase H. The cleavage products were analyzed by denaturing polyacrylamide/8 M urea gel electrophoresis and are presented schematically on the *right*. Cleavage sites are indicated by *arrowheads* and labeled products are shown in *black*. ODN Co, annealing to sequences downstream of the PPT, served as a control. **d** Following the experimental procedure shown at the *top*: Freshly thawed ODN A or ODN Co (250 nM), or ODN A stored for 102 days at 37 °C in PBS were incubated with replication-competent HIV-1 particles (1 × 10^9^) at 37 °C for 6 h in cell culture medium. Jurkat 1G5 T cells were infected with the mixtures overnight and HIV-1 p24 antigen in the supernatant was detected at 3–14 days post infection. Two-way ANOVA followed by Bonferroni posttest was used for statistical evaluation. ODN A-mediated inhibition (as compared to buffer alone) was highly significant (*p* < 0.001) at day 7–14 post infection

The antiviral effect of ODN A has already been extensively investigated in vitro and in vivo [11–13, 15, 16, 29]. These data suggested that ODN A might indeed be a valuable component of future vaginal antiviral microbicides. However, further successful microbicide development requires that the antiviral agent possesses outstanding drug stability. To test such properties, we kept ODN A for extended time periods in phosphate-buffered saline (PBS) (Fig. 2a) or in water (H_2_O) (Fig. 2b) at 37 °C and subsequently analyzed the samples by native PAGE and SYBR Green II staining. Surprisingly, ODN A formed a prominent high molecular structure as visualized by non-denaturing polyacrylamide gel electrophoresis, and no degradation products of ODN A multimers or monomers were observed over the entire period of up to 102 days in PBS at 37 °C (Fig. 2a). In water, the ODN A complex displayed compound stability for up to ~2 months, although subsequently some degradation was observed (Fig. 2b), indicating that the ionic PBS components play an important role in stabilizing the high molecular structures of ODN A. Obviously, in water, the high-ordered structures are dissolved over time and additional signals appeared with higher electrophoretic mobility. Nevertheless, even after 88 days of incubation, some of the ODN A complex was still detectable.

After demonstrating high stability of the ODN A hyperstructure, we next investigated the effect of ODN A on RNase H after long-term storage. First, we analyzed ODN A-mediated cleavage by RNase H using synthetic radioactive 5′ end-labeled in vitro transcribed HIV-1 RNA containing the extended PPT. Freshly thawed ODN A (positive control), as well as ODN A stored for 73 or 102 days in PBS cleaved the PPT RNA template almost entirely, resulting in defined fragments (Fig. 2c). Interestingly, ODN A stored in H_2_O was comparably active in this in vitro assay. It is noted that a fragment with lower mobility appears in the ODN Co-specific reaction. This reflects the fact that ODN Co binds to sequences downstream of the PPT.

Next, to analyze the antiviral activity of ODN A, HIV-1 particles were pre-incubated for 6 h together with sample buffer, 250 nM of ODN A or ODN Co at 37 °C, followed by overnight infection of Jurkat 1G5 T cells (see experimental design outlined in Fig. 2d). Sixteen hours later, culture supernatants were replaced with fresh medium that was supplemented with the respective ODNs. Every 3–4 days, supernatants were collected to monitor HIV-1 particle release by HIV-1 p24 antigen ELISA. Cells were reseeded into fresh cell culture medium, again supplemented with the respective ODNs. Uninfected cells were included as a negative control, whereas ODN Co and the sample without ODN (Buffer) served as positive controls. No HIV-1 particles were detected in the supernatant of cells infected in the presence of freshly thawed ODN A (green points), nor in the presence of long-term stored ODN A (red points) (Fig. 2d). In contrast, the amount of p24 HIV-1 in the supernatant increased over time in the samples without ODN (Buffer, grey points) or in the presence of ODN Co (black points), indicating successful infection of the 1G5 Jurkat T cells (Fig. 2d). Thus, despite storage for several months at 37 °C, ODN A showed efficient RNase H-assisted cleavage of in vitro transcribed PPT-containing RNA as well as high antiviral activity in cell culture infection experiments.

### The lubricant K-Y Jelly does not influence the stability or antiviral activity of ODN A

**Fig. 3:**
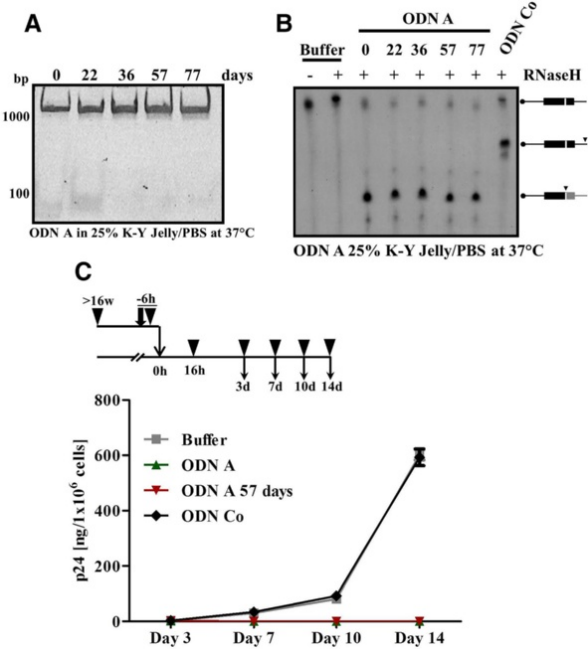
The lubricant CMC (K-Y Jelly) does not influence the stability and antiviral activity of ODN A. **a** ODN A (8 μM) was incubated for the indicated days in 25 % K-Y Jelly (in PBS) at 37 °C. The samples were analyzed by 10 % non-denaturing polyacrylamide gel electrophoresis. **b** Analysis of ODN A-triggered, RNase H-mediated cleavage of PPT RNA in vitro. Assays were performed as described in Fig. 2c. Cleavage sites are indicated by *arrowheads* and the labeled products are indicated in *black*. **c** Following the experimental procedure shown at the *top*: ODN A pre-incubated in 25 % K-Y Jelly/PBS for 57 days, freshly thawed ODN A, or ODN Co (both 250 nM) were incubated with replication-competent HIV-1 virions (1 × 10^9^) at 37 °C for 6 h in cell culture medium. Jurkat 1G5 T cells were subsequently infected overnight. Virus replication was monitored by HIV-1 p24 antigen ELISA of culture supernatants at day 3–14 post infection. Two-way ANOVA followed by Bonferroni posttest was used for statistical evaluation. ODN A-mediated inhibition (as compared to buffer alone) was significant (day 7, *p* < 0.05; day 10 and 14, *p* < 0.001)

Next, we analyzed whether a potential carrier for microbicide application in humans, such as the established lubricant carboxymethylcellulose CMC (K-Y Jelly) [15, 30–33], affects ODN A’s stability or antiviral activity. As before, ODN A was stored for extended periods of time in 25 % K-Y Jelly/PBS at 37 °C. Subsequent analyses revealed that the presence of K-Y Jelly did not negatively affect ODN A degradation (Fig. 3a), nor RNase H-mediated PPT RNA cleavage and the antiviral activity of ODN A (Fig. 3b and c). Moreover, ODN A exposed to 25 % K-Y Jelly for up to 57 days did not lose its pronounced anti-HIV-1 activity (Fig. 3c). Thus, personal water-based lubricants used in microbicide formulas, such as K-Y Jelly, apparently do not interfere with ODN A activity.

### The significant thermostability of ODN A depends on it forming G-quadruplex-based DNA structures

The nature of the observed ODN A-specific high molecular weight structures was particularly interesting. It is known that oligonucleotides containing several guanosines can form four-stranded, non-canonical DNA structures, called G-quadruplexes [34, 35]. Within a quadruplex, tetrads of guanosines form by interacting via additional hydrogen bonds, known as Hoogsteen base pairing [22, 24]. A unique feature of quadruplex structures is selectively increased stability in the presence of potassium ions that are complexed by the guanosine tetrads. Since the passenger strand of ODN A indeed contains multiple guanosines (Fig. 1b), we hypothesized that these residues account for the observed high ordered structures [36].

**Fig. 4:**
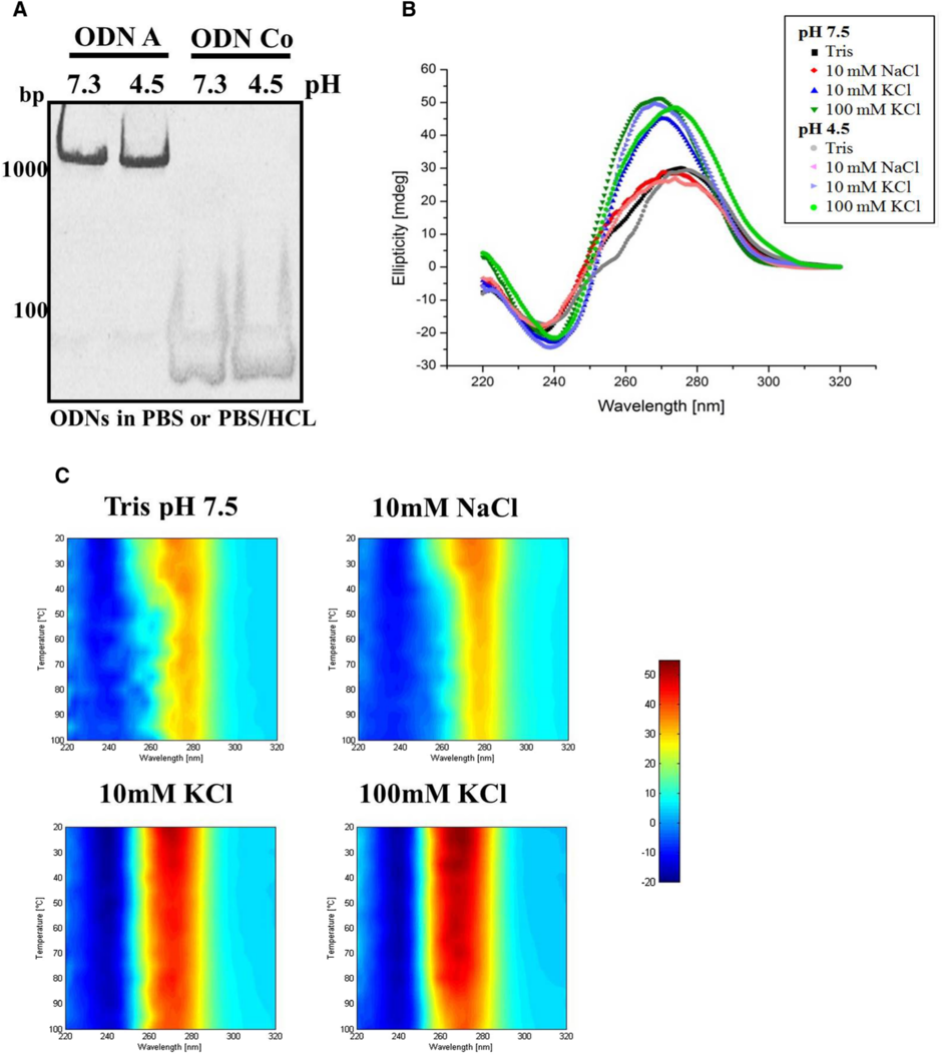
Structural analysis of ODN A. **a** The indicated ODNs (8 μM) were incubated for 1 h in PBS pH 7.4 or in PBS/HCl pH 4.5 and analyzed by native polyacrylamide gel electrophoresis. **b** CD spectra of ODN A were recorded at different pH and different ionic concentrations. **c** CD spectra of ODN A were recorded between 20 and 100 °C using various ionic concentrations. Wavelengths are indicated on the *right*. Ellipticity: *dark red* = 55 mdeg; *dark blue* = −20 mdeg

We analyzed the secondary structure of ODN A in different buffer conditions by CD spectroscopy (Fig. 4b). In buffer lacking potassium the spectra reassembled the expected spectra of B-DNA in a hairpin structure [37]. In contrast, in potassium-containing buffer ODN A produced a strong absorption maximum at 270 nm and a minimum at 240 nm. The fact that potassium stabilizes G-quadruplexes and these CD data resemble classical spectra of parallel G-quadruplexes provides strong evidence for quadruplex formation of ODN A. Interestingly, pH 4.5 did not influence the formation of the aggregates (Fig. 4a and b).

These spectra, together with the data obtained by non-denaturing PAGE suggest that ODN A forms high molecular weight complexes, most likely so-called DNA “frayed wires” [22, 23, 25]. Frayed wires are apparently formed intermolecularly by deoxyoligonucleotides comprising elongated stretches of guanosines in combination with adenosine or thymidine-rich sequences. The multimeric DNA complex is then mediated by intermolecular G-quadruplex formation, resulting in a G-core with single-stranded adenosine-/thymidine-rich sequences protruding from the stem [22, 23, 37].

G-based high ordered structures are frequently characterized by increased solubility and high thermostability [25, 37]. To investigate this we performed thermal denaturation CD spectroscopy between 20 and 100 °C in different buffer conditions (Fig. 4c). Interestingly, ODN A formed aggregates that were stable up to 100 °C. Increasing potassium concentrations dramatically stabilized these structures (Fig. 4c).

In conclusion, ODN A forms potassium-dependent, multimeric hyperstructured complexes that are pH-independent and characterized by high thermostability.

### The G-quadruplex-based structure of ODN A contributes to its antiviral activity

It has been previously shown that the strong antiviral activity of ODN A is due to its activation of the viral RNase H [11–13, 15, 16, 29]. However, the finding that ODN A forms G-based hyperstructures raised the question of whether these structures can interfere with HIV infectivity [38–41]. Therefore, we now included ODN G in our experimental set-up. Compared to ODN A, ODN G has nucleotide exchanges specifically in its antisense strand (Fig. 1b), which allows formation of DNA hyperstructures without binding to the HIV-1 PPT.

**Fig. 5:**
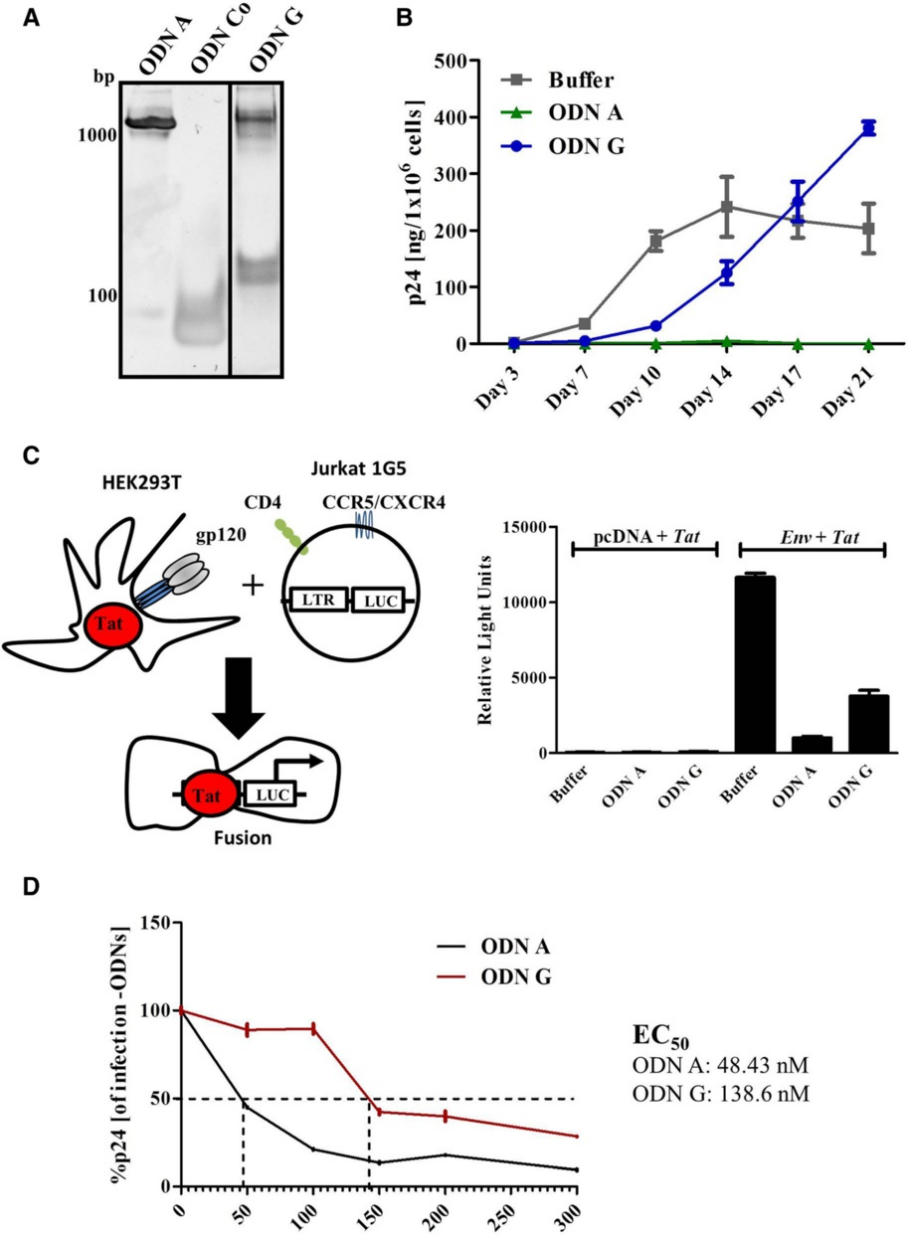
The formation of G-based hyperstructures contributes to ODN A’s antiviral activity. **a** ODN A, ODN Co and ODN G (8 μM) were incubated for 1 h in PBS and analyzed by native polyacrylamide gel electrophoresis. **b** ODN A, ODN Co, ODN G (250 nM) or buffer alone were incubated together with HIV-1 particles (1 × 10^9^) for 6 h at 37 °C. Following infection of Jurkat 1G5 T cells, release of p24 antigen was detected over time in the culture supernatants (day 3–21 post infection). Two-way ANOVA followed by Bonferroni posttest was used for statistical evaluation. ODN A-mediated inhibition (as compared to buffer alone) was highly significant (*p* < 0.001) at day 10–21 post infection. ODN G-mediated inhibition (as compared to buffer alone) was highly significant (*p* < 0.001) at day 10, 14 and 21 post infection. **c** Following the experimental design depicted at the left, HEK293T cells were transfected with plasmid vectors expressing HIV-1 Env and Tat, or the parental vector (pcDNA) as a negative control. At 24 h post transfection, Jurkat 1G5 reporter T cells, which were pre-incubated for 1 h in 500 nM ODN or buffer alone (negative control) were added to the HEK293T cell cultures for another 24 h. Jurkat 1G5 T cell-derived luciferase signals were subsequently measured, indicating successful cell fusion. One-way ANOVA followed by Dunnett’s Multiple Comparison Test was used for statistical evaluation. ODN A-and ODN G-mediated cell fusion (as compared to buffer alone) was highly significant (*p* < 0.001). **d** Different concentrations of ODN A or ODN G were pre-incubated with HIV-1 as before. Subsequently, Jurkat 1G5 T cells were infected and HIV-1 p24 antigen release was detected at day 7 post infection. EC_50_ values were calculated using GraphPad PRISM (Graphpad Software, Inc, USA) software

Complex formation was visualized by native PAGE. Whereas ODN Co migrated as a monomer, ODN A and ODN G formed comparable high molecular structures (Fig. 5a). When HIV-1 particles were pre-incubated with ODNs as before, ODN A again demonstrated complete inhibition of *de novo* infection, whereas ODN G-treatment resulted in delayed accumulation of viral particles in the culture supernatants, characterized by high viremia towards the end of this experiment (day 17–21) (Fig. 5b). More rapid development of viremia was clearly observed in the absence of ODNs (Buffer), somewhat declining towards the end of the experimental timeframe (day 17–21). This presumably reflects the fact that early and strong virus replication may cause pathogenic effects in this cell culture.

Guanosine-based hyperstructures have been reported to decrease the binding of HIV-1 to host cell membranes [39, 40]. Therefore we next employed an established cell culture-based fusion assay system [42] to test ODN A’s or ODN G’s capacity to block the interaction of HIV-1 Env with the cellular CD4 surface molecule. HEK293T cells were cotransfected with vectors expressing HIV-1 Tat and Env, or with the parental pcDNA plasmid as a negative control. At 24 h post transfection, Jurkat 1G5 T cells, which were pre-incubated in buffer (additional control) or in 500 nM of ODNs, were added to the HEK293T cultures for a further 24 h. Note that Jurkat 1G5 T cells contain a firefly luciferase expression cassette under the control of an HIV-1 LTR promoter; thus, Env-CD4 interaction and subsequent cell fusion enables Tat-mediated luciferase expression (schematically depicted in Fig. 5c, left panel). As expected, this assay clearly revealed that both ODN A and ODN G diminished the interaction of membrane-bound viral Env and cellular CD4 molecules (Fig. 5c, right panel), confirming that G-based structures can per se interfere with HIV infectivity [38–41].

To further evaluate the antiviral potency of ODN A and ODN G we next defined the EC_50_ values for both agents. Jurkat 1G5 T cells were infected overnight with HIV-1, which was pre-incubated for 6 h at 37 °C in different concentrations of ODN A or ODN G. At 7 days post infection, amounts of viral particles in the supernatant were determined by HIV-1 p24 antigen ELISA and the signal obtained in the sample without ODNs was arbitrarily set to 100 %. ODN A displayed an EC_50_ value of 48.43 nM, whereas ODN G had an EC_50_ value of 138.6 nM (Fig. 5d). Clearly, the increased antiviral potency of ODN A can be explained by its dual function, not only interfering with virus uptake via its G-tetrads and possibly DNA frayed wire structure, but also triggering viral genome cleavage by prematurely activating the viral RT/RNase H.

### ODN A activity in the presence of human semen and semen-derived amyloid fibrils

**Fig. 6:**
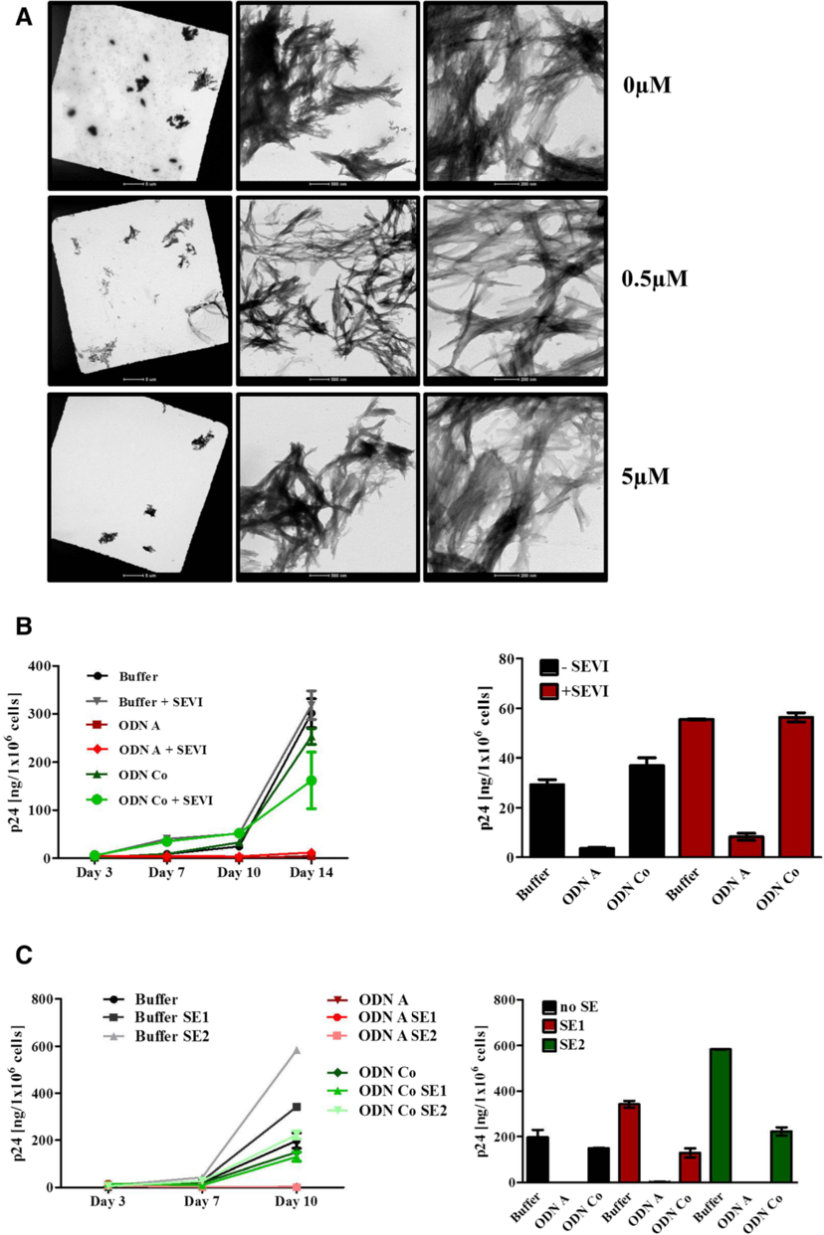
ODN A does not accelerate amyloid fibril formation and maintains its antiviral activity in the presence of human semen and SEVI. **a** PAP_248-286_ peptides were incubated at 37 °C for 72 h in the presence or absence of 0.5 or 5 μM ODN A. Afterwards, amyloid fibrils were detected by negative-stain transmission electron microscopy. *Scale bars* indicate 5 μm, 500 nm or 100 nm, left to right. **b** HIV-1 particles (5 × 10^8^) were incubated for 6 h ± 250 nM ODNs and ± 100 μg/ml SEVI. Jurkat 1G5 T cells were infected overnight with the respective mixtures and HIV-1 p24 antigen release into the supernatants was monitored by ELISA at the indicated days. The p24 antigen level on day 7 post infection is depicted as a bar chart on the *right*. Two-way ANOVA followed by Bonferroni posttest was used for statistical evaluation. ODN A-mediated inhibition (+/- SEVI; as compared to buffer alone) was highly significant (*p* < 0.001) at day 14 post infection. **c** Analysis of HIV-1 infectivity essentially as described in panel b. Prior to *de novo* infection, HIV-1 particles were incubated for 6 h ± 250 nM ODNs and 2 different human semen samples. Statistics were evaluated as before. ODN A-mediated inhibition (+/- SE1 or SE2; as compared to buffer alone) was highly significant (*p* < 0.001) at day 10 post infection

Despite high antiviral potency and pronounced thermostability, antiviral compounds may fail as microbicides in the presence of semen, which has been shown to significantly enhance HIV infectivity by forming semen-derived amyloid fibrils [18, 19, 43, 44]. In fact, several polyanionic candidate microbicides have been reported to accelerate semen-derived fibril formation [20], thereby further enhancing HIV infection. Since ODN A is also a polyanionic compound, we next examined its effect on the in vitro formation of SEVI, which is formed by peptides proteolytically released from prostatic acid phosphatase (PAP) [18]. Synthetic PAP-derived peptides (PAP_248-286_) were incubated for 72 h at 37 °C in the presence of high concentrations of ODN A. In turn, formation of amyloid fibrils was visualized by negative-stain transmission electron microscopy (TEM). No acceleration of amyloid fibril formation was observed in samples containing 0.5 or 5 μM ODN A when compared to a sample without ODN (Fig. 6a).

In addition to enhancing HIV infectivity, human semen may also impair the efficacy of microbicides [21]. Therefore, the antiviral activity of ODN A in the presence of synthetic SEVI fibrils or human semen (SE) samples was analyzed directly. HIV-1 particles were pre-incubated at 37 °C for 4 h together with 250 nM ODNs alone, ODNs plus 100 μg SEVI, or buffer alone (Fig. 6b). Likewise, viral particles were pre-incubated with combinations of ODNs and different human semen samples (Fig. 6c). Subsequently, 5 × 10^5^ Jurkat 1G5 T cells were infected with the respective mixtures. Sixteen hours later, the cells were reseeded in fresh culture medium (supplemented with ODNs or sample buffer only). Every 3–4 days, supernatants were collected to determine the amount of released viral particles by HIV-1 p24 antigen ELISA. As expected, SEVI clearly increased HIV-1 infectivity as shown at day 7 and day 10 post infection (Fig. 6b). However, ODN A displayed strong antiviral activity, even in the presence of SEVI (Fig. 6b). Comparable results were observed when the synthetic SEVI was replaced by human semen (from two different donors) (Fig. 6c).

In summary, these data showed that ODN A does not accelerate amyloid fibril formation and demonstrates high antiviral efficacy, even in presence of SEVI or human semen.

## Discussion

Novel classes of antiretroviral microbicides are considered to be important tools to halt sexual transmission of HIV, particularly in countries where social conventions hamper the use of condoms and access to antiretroviral medicines is limited. Various different delivery systems are currently used for microbicide application. In the majority of current clinical trials, microbicides are formulated as vaginal gels, vaginal tablets, intravaginal rings or long-acting injectables [45]. Moreover, microbicidal antiretroviral activity may be combined with contraception or with drugs targeting, for example, HSV-2 or other sexual transmitted diseases [45–47].

Clearly, user adherence to novel microbicides, especially in the Third World, depends not only on antiviral efficacy, but also on the easiness of application and storage. Therefore, the main goal in this study was to investigate the activity, and particularly the drug stability of the antiretroviral agent ODN A. This novel and advanced oligonucleotide-based compound has been previously shown to target the highly conserved extended PPT of HIV-1, leading to premature activation of the RT/RNase H complex, resulting in degradation of the viral RNA genome [11–16, 29].

Here, we successfully demonstrated the pronounced and unexpected stability of ODN A, without any reduction of its antiviral potency, even when stored for several months at 37 °C. Furthermore, the lubricant K-Y Jelly [30], a likely component of a pharmaceutical microbicide formulation, did not affect the stability or antiviral potency of ODN A. This suggests that the inclusion of ODN A into a future long-acting microbicide (i.e. delivered by intravaginal rings, injectables or gels) will probably not require additional drug modifications to improve drug stability.

Another important aspect in microbicide development is assessing the potential effects of human semen on the efficacy of antiviral agents. The major component of semen is a coagulum containing spermatozoa and semenogelin proteins [48, 49]. These proteins are proteolytically cleaved into smaller peptide fragments by prostate-specific antigen (PSA), generating cationic amyloid fibrils. These fibrils are known to facilitate HIV infection by enhancing the attachment of virions to cells, and perhaps by altering the immunological environment within the female mucosa [18, 19, 43, 44, 50, 51]. Importantly, such amyloid fibrils can also decrease the antiviral efficacy of antiviral drugs, especially polyanionic agents targeting the virus itself [21]. The exact reason for this is unknown, but neutralization of the drugs’ negative charge, or competitive binding to the viral envelope seems to be involved [21, 52, 53].

Moreover, polyanionic compounds such as cellulose sulfate, carrageenan and PRO2000, potentially acting as HIV-1 entry inhibitors, when previously analyzed in microbicide clinical trials, in some cases unexpectedly resulted in increased infection rates [54, 55]. These rather sobering results were linked to enhanced formation of semen-derived fibrils by the microbicide candidates [20]. Fortunately, the TEM data obtained in this study demonstrated that, although negatively charged, ODN A does not accelerate amyloid fibril formation. More importantly, neither synthetic amyloid fibrils (i.e. SEVI) nor human semen negatively affected ODN A’s antiviral activity in infection assays.

ODN A has a length of 54 nucleotides and forms a hairpin-like structure with an antisense strand binding to the HIV-1 PPT and a partially complementary passenger strand for stabilization [12, 29]. The passenger strand contains a guanosine-rich stretch that can potentially form G-quadruplex-based larger hyperstructures, so-called G-wires or DNA frayed wires [22, 23, 25, 56]. Indeed, the CD spectra presented here displayed typical characteristics of G-quadruplex-based structures with parallel strand orientation indicated by a strong maximum at 270 nm and minimum at 240 nm, particularly in the presence of potassium. The secondary structure of ODN A is very thermostable and pH-independent. The spectra together with the high-ordered structures seen on native PAGE indicate that ODN A aggregates to DNA frayed wires formed by deoxyoligonucleotides containing runs of guanosines [22, 25].

Obviously, the formation of G-based quadruplexes would explain the surprisingly high stability of ODN A-based hyperstructures, which combined with its intrinsic solubility, is highly advantageous for further microbicide development. It is known that related DNA aptamers and G-quadruplexes can diminish HIV infectivity by interfering with the binding of viral particles to host cells, or by inhibiting reverse transcription or HIV integration [38–41, 57]. To determine whether the passenger strand contributes to ODN A’s significant antiviral potency we used ODN G, a variant unable to recognize the PPT. Indeed, this oligonucleotide also formed high molecular structures, although its antiviral potency was much lower compared to ODN A. Clearly, the mechanisms of how ODN A molecules enter living cells (or even viral particles) remains to be elucidated. However, it was previously shown that ODNs can enter cells in large quantities, when HIV-1 particles are present [58].

Due to its dual mode of action, ODN A appears to be optimally positioned to act as a powerful antiviral agent. Its target, the PPT, occurs in 69 % of primary HIV-1 isolates [59] and, consequently, several patient-derived viruses, including antiretroviral drug-resistant viruses, have been shown to be fully susceptible to ODN A-mediated inhibition [12, 17]. Nonetheless, viral diversity of some HIV subtypes or strains may negatively impact on ODN A’s antiviral efficacy, an effect that may be overcome by applying a mixture of ODN A variants.

ODN A not only forms G-based DNA hyperstructures, thereby targeting viral entry, but also prematurely activates the viral RT/RNase H complex by simulating naturally occurring DNA-RNA hybrids, leading to degradation of the viral RNA genome before reverse transcription can occur. Since ODN A is an extraordinary stable compound with high anti-HIV properties, even in the presence of semen or lubricant, it represents an ideal candidate for further development as an antiviral microbicide. It has to be stated, however, that the in vitro models used in the present study do not exactly reproduce the conditions occurring in vivo (e.g. mimicking the mucosal environment etc.). Therefore, future studies will focus on the analysis of GMP-produced ODN A in appropriate animal models. Particularly, these studies will analyze the occurrence of potential drug-related toxicities and the potential development of antiviral resistance.

## Conclusions

Globally, HIV is primarily transmitted by sexual intercourse and predominantly infects people in developing countries. Therefore, advanced vaginal microbicides are highly needed to provide female-controlled methods of HIV prevention in such environments. Here, we report very high stability, solubility and antiviral potency of ODN A, a 54-mer oligonucleotide that forms G-based DNA hyperstructures. In particular, ODN A demonstrates antiviral activity in the presence of human semen, or an approved lubricant for human use, and may therefore be a valuable component of future vaginal microbicides.

## Abbreviations

ART, antiretroviral therapy; CD, circular dichroism; ELISA, enzyme-linked immunosorbent assay; LTR, long terminal repeat; NNRTI, non-nucleoside reverse transcriptase inhibitor; NRTI, nucleoside-analogue reverse transcriptase inhibitor; ODN, oligodeoxynucleotide; PAGE, polyacrylamide gel electrophoresis; PAP, prostatic acid phosphatase; PBS, phosphate-buffered saline; PPT, polypurine tract; PSA, prostate-specific antigen; SE, semen; SEVI, semen-derived enhancer of virus infection; TEM, transmission electron microscopy
